# Supplementary material for: The Contribution of Antimicrobial Peptides to Immune Cell Function: A Review of Recent Advances
Source: Pharmaceutics. 2023 Sep 4;15(9):2278. doi: 10.3390/pharmaceutics15092278 (PMC10535326; doi:10.3390/pharmaceutics15092278)
Supplement: Supplementary file 1 [file pharmaceutics-15-02278-s001.zip › pharmaceutics-2541745-supplementary.pdf]

pharmaceutics

MDPI

Supplementary Table S1. 96 AMPs have been developed as drug candidates for the peptide drug market

DRAMP ID	Peptide Name	Biological Activity	Description	Medical use	Stage of development use	Company
DRAMP 18057	MSI-78(Pexiganan, Locilex)	Antibacterial	Pexiganan is a broad-spectrum synthetic analogue of the African frog peptide magainin.	Infected diabetic foot ulcers	Phase III(Failure)	Genaera (Plymouth, PA)
DRAMP 18058	Daptomycin(Cubicin, LY-164032)	Antibacterial	Daptomycin is a cyclic lipopeptide antibiotic used to treat complicated skin and skin structure infections by susceptible Gram-positive bacteria and bacteremia due to Staphylococcus aureus.	Treatment of complicated skin and skin structure infections (cSSSI), Staphylococcus aureus bloodstream infections (bacteremia)	In market	Cubist Pharmaceuticals LLC (Merck & Co.)
DRAMP 18059	Iseganan(IB-367)	Antibacterial, Antifungal	Iseganan is a synthetic analogue of Protegrin I (IBL-367, the pig protegrin derivative), extracted from porcine leukocytes.	①Prevention of Ventilator-Associated Pneumonia; ② Prevention of oral complications caused by radiation therapy for head and neck cancer.	Phase III(Failure)	Intrabiotics Pharmaceuticals Inc.(Mountain View, CA)
DRAMP 18060	p2TA(AB 103, Reltecimod)	Antibacterial	Reltecimod is under investigation in clinical trial NCT02469857 (Phase III Efficacy and Safety Study of AB103 in the Treatment of Patients With Necrotizing Soft Tissue Infections).	Necrotizing soft tissue infections	Phase III	Atox Bio Ltd.
DRAMP 18061	Histatin	Antifungal(fungal cell membrane,mitochondrion)	using a variant of histatins, which are naturally occurring cationic peptides in saliva	antimicrobial-peptide-containing mouth wash for the treatment of oral candidiasis (gingivitis and periodontal diseases)	Phase II-III	Demgen and Dow Pharmaceutical Sciences;licensed to the Vancouver, Canada, company Pacgen
DRAMP 18062	Ramoplanin(NTI-851)	Antibacterial	Ramoplanin(NTI-851) is a macrocyclic glycolipodepsipeptideproduced by Actinoplanesspp. being developed by Nano-therapeutics.It exhibits bactericidal activity by blocking the cell wall peptidoglycansynthesis of gram-positive bacteria. Recently, the phase III clinicalstudy of the peptide was initiated for the oral treatment ofvancomycin-resistantenterococcus(VRE) colonization, as well as the phase II trial against Clostridium difficile.	Treatment of bacterial infections.	Phase III	Nano-therapeutics
DRAMP 18063	P113	Antifungal	a 12 amino acid fragment of histatin 5	Reduce gum bleeding, gingivitis and plaque	Phase II	Demgen
DRAMP 18064	P113D	Antifungal	a 12 amino acid fragment of histatin 5	an inhalation treatment for Pseudomonas	Unknown	Demgen

				aeruginosainfections in cystic fibrosis patients		
DRAMP 18067	Dalbavancin (BI397, Dalvance, Xydalba)	Antibacterial	Dalbavancin is a semisynthetic lipoglycopeptide and derivate of teicoplanin. It is an antibacterial used to treat acute bacterial skin and skin structure infections (ABSSSI) caused by susceptible strains of Gram-positive bacteria.	Acute bacterial skin infections, Osteomyelitis and septic arthritis(Phase IV)	In market	Allergan (formerly Actavis and Durata Therapeutics)
DRAMP 18068	hLF1-11(Human lactoferrin 1-11)	Antibacterial, Antifungal	hLF1-11 was significantly more effective than the full length protein or the peptide representing the second cationic domain. As with other antimicrobial peptides, hLF1-11 shows poor antimicrobial activity under physiological conditions in vitro, but it is highly effective in vivo against infections due to a variety of microorganisms, including Gram negative and Gram positive bacteria and fungi. The objective is to develop hLF1-11 for the treatment of fungal and bacterial infections that develop during neutropenia that results from myeloablative therapy to prepare for a haematopoietic stem cell transplant(HSCT) formerly referred to as bone marrow transplant.	Bacteraemia and fungal infections in immunocompromized haematopoetic stem cell transplant recipients	Phase I-II	AM-Pharma Holding BV
DRAMP 18069	rBPI21(Neuprex)	Antibacterial, Antiendotoxin(bacterial membranes)	a human bactericidal permeability protein derivative	meningococcaemia;prophylactic treatment of infectious complications from post-traumatic bleeding (in 842 patients)	Phase III(Failure)	Xoma Ltd(Berkeley, CA, USA)
DRAMP 18070	XMP 629(HY-P2170; CS-0109623)	Unknown	extracted from human	Impetigo and acne rosacea	Phase III	Xoma Ltd(Berkeley, CA, USA)
DRAMP 18071	Mycoprex	Unknown	extracted from insects	Fungal infections	Phase III	Xoma Ltd.
DRAMP 18072	Heliomocin variants(ETD151)	Unknown	an antifungal 44 amino acid peptide, variant of a natural peptide from lepidopteran Heliothis virescens	Systemic fungal infections in immunocompromised patients and multi-resistant bacterial infections	Preclinical	EntoMed SA
DRAMP 18073	PG-1	Unknown	Protegrin Class	Treatment of peritoneal infections caused by P. aeruginosa and S. aureus.Treatment of pneumonia	Unknown	Intrabiotics
DRAMP 18074	CSA-13	Unknown	cationic steroid (ceragenin) that mimics host-defense peptides	Anti-infective	Preclinical	Ceragenix(Denver)
DRAMP 18075	HB-50	Unknown	synthetic natural peptide mimetic of cecropin	Anti-infective	Preclinical	Helix Biomedix(Bothell, Washington, USA)
DRAMP 18076	HB-107	Unknown	19-amino-acid fragment of cecropin B	Wound healing	Preclinical	Helix Biomedix(Bothell,

						Washington , USA)
DRAMP 18077	HB-1345	Antibacterial	Synthetic Lipohexapeptide	acne,broad-spectrum antibiotic	Pre-Phase I	Helix Biomedix (Bothell, Washington , USA).
DRAMP 18078	HB1275	Antifungal	A lipohexapeptide with potent antifungal activity against yeast and filamentous fungi	Trichophyton infections	Preclinical	Helix Biomedix (Bothell, Washington , USA).
DRAMP 18079	Mersacidi n	Unknown	Mersacidin is a type-B lantibiotic containing 3-methyllanthionine and S-(2-aminovinyl)-3-methylcysteine residues and four intra-chain thioether bridges. It is obtained from Bacillus sp. HIL Y-85,54728 and is active in vivo against methicillin-resistant Staphylococcus aureus (MRSA). It has a role as a metabolite and an antibacterial agent. It is a type B lantibiotic and a macrocycle. It is a tautomer of a mersacidin zwitterion.	Gram-positive infections,MRSA	Preclinical	Novacta Biosystems Ltd.(Hatfiel d, England)
DRAMP 18080	Plectasin	Antibacterial	fungal defensin	Systemic antiâ€"Gram positive,especially pneumococcal and streptococcal infections	Phase I	Novozymes A/S(Bagsva erd, Denmark)
DRAMP 18081	PAC113	Antifungal	based on the active segment of histatin 5 protein found in human saliva	Oral candidiasis in HIV seropositive patients	Phase IIb	Pacgen(Van couver, BC, Canada)
DRAMP 18082	Peptidomi metics	Unknown	derived from the arylamide, calixarene, hydrazide and salicylamide series	Anti-infectives; antimicrobial polymers and coating materials	Preclinical	Polymedix(Philadelphia)
DRAMP 18083	CZEN-002(Mela ntropin, (CKPV)2)	anti-candidal	CZEN-002 is a novel, non-azole anti-fungal synthetic octapeptide, derived from alpha-Melanocyte-Stimulating Hormone (a-MSH). CZEN-002 modulates inflammatory and immune responses. It has also been shown to kill Candida albicans (C. albicans), a single-celled fungal organism that causes a variety of infections, including vaginitis. This organism can invade tissues and produce fatal infections in individuals with compromised immune systems such as those suffering from HIV/AIDS or undergoing organ or bone transplants. The antimicrobial activity of CZEN-002 is unique in that it does not depend on direct damage to the microbial membrane. It appears that CZEN-002 works on a receptor in yeast that has yet to be identified.	GPB, GNP, Candida. Yeast regulatory mechanisms, Interference by cAMP induction, anti-inflammatory.	Phase IIb	Zengen(Wo odland Hills, CA, USA)
DRAMP 18084	Talactofer rin alpha(Lact	Anticancer	Talactoferrin alfa is a novel immunomodulatory 80 kD protein with demonstrated oral anti-tumor properties.	Combined with chemotherapy in the first-line treatment of advanced non-small cell lung	Phase III	Agennix

	oferroxin-A)		Lactoferrin, a protein found in breast milk is developed by Agennix. It increases body's immune power and also works as a natural antioxidant, helping to control cell and tissue damage caused by oxidation.	cancer;Topical treatment in diabetic neutropathic ulcers		
DRAMP 18085	CSA(cationic steroid antibiotics)	Unknown	The CSAs share structural features in common with the AMPs that form part of the bodys innate immune system.	Anti-infective,including multi-drug resistant organisms such as pseudomonas, MRSA and vancomycin-resistant S. aureus	Preclinical	Ceragenix
DRAMP 18086	Telavancin(TD-6424)	Antibacterial	Telavancin is a semi-synthetic derivative of vanocymycin that has bactericidal activity against Methicillin-resistant Staphylococcus aureus (MRSA) and other gram-positive bacteria.	Osteomyelitis, Bacterial infections (I)	In market	Clinigen Group plc/Innoviva Inc./Pendopharm/Theravance Biopharma Inc./University of Illinois
DRAMP 18087	D2A21(Demegal, Provena)	Antibacterial	a 22-residue αhelix peptide	Burn infection, skin infection with multidrug-resistant pathogens	Phase III	Demegen
DRAMP 18088	EA-230	Unknown	Oligopeptide fragment fromb-hCG(4-mer, LQGV)	Anti-inflammatory. Sepsis and renal failure protection.	Phase II	Exponential Biotherapies
DRAMP 18089	Amphiphilic arylamide polymers	Unknown	No more description	Anti-infective drug and antimicrobial polymers and coating materials	Preclinical	Polymedix
DRAMP 18090	LL-37(Cathelicidin, ropocamptide, Bac4, CAS001)	Antibacte-rial(Mono-cyte,T-cell, neutrophil chemotaxis)	Human (neutrophils and epithelial cells)	Hard-to-heal venous leg ulcers	Phase I/II	Inimex Pharmaceuticals
DRAMP 18091	PTX001	Unknown	potent anti-angiogenic agents	Broad-spectrum antimicrobial;Antiendotoxin	Preclinical	PepTx
DRAMP 18092	PTX002	Antibacterial(bacterial membranes)	33-mer peptide;	Broad-spectrum antimicrobial;Antiendotoxin	Preclinical	PepTx
DRAMP 18093	PTX004	Unknown	potent anti-angiogenic agents	Broad-spectrum antimicrobial;Antiendotoxin	Preclinical	PepTx
DRAMP 18094	PTX005	Antibacterial(bacterial membranes)	12-mer peptide	Broad-spectrum antimicrobial;Antiendotoxin	Preclinical	PepTx
DRAMP 18095	PTX006	Unknown	N-acylated analog of PTX005	Broad-spectrum antimicrobial;Antiendotoxin	Preclinical	PepTx
DRAMP 18096	PTX007	Unknown	a nonpeptidic structural analog of PTX005	Broad-spectrum antimicrobial;Antiendotoxin	Preclinical	PepTx
DRAMP 18150	PTX008	Unknown	potent anti-angiogenic agents	Broad-spectrum antimicrobial;Antiendotoxin	Preclinical	PepTx

DRAMP 18151	Glutoxim(NOV-002)	Anticancer	Glutathione Disulfide NOV-002 is a stabilized formulation of disodium glutathione disulfide (GSSG; oxidized glutathione) and cisplatin (1000:1) with potential chemoprotective and immunomodulating activities. Mimicking endogenous GSSG, glutathione disulfide NOV-002 acts as a competitive substrate for gamma-glutamyl-transpeptidase (GGT), which may result in the S-glutathionylation of proteins, predominantly actin, a redox stress on endoplasmic reticulum (ER), and ER stress-induced apoptosis; S-glutathionylation may be stimulated by reactive oxygen species (ROS) liberated by a glutathione disulfide NOV-002-induced increase in GGT activity. Glutathione disulfide NOV-002 may also induce phosphorylation of proteins such as ERK and p38, two kinases that play critical regulatory roles in cell proliferation and apoptosis. The cisplatin component of this agent does not provide an effective therapeutic concentration of cisplatin in vivo.	Tuberculosis, non small cell, lung cancer	Phase III	Pharma BAM/Novelos
DRAMP 18152	IMX942(Inimex)	Broad spectrum	Synthetic cationic host defense peptide,derivative of IDR-1 and indolicidin	Treatment for chemotherapeutic induced neutropenia, nosocomial infections	Phase II	Inimex
DRAMP 18153	Opebacan	Antibacterial, Antiviral	21-amino-acid peptide derivative of bactericidal/permeability-increasing protein	Endotoxemia in hematopoetic, stem cell transplant, recipients	Phase I/II	Xoma
DRAMP 18154	XOMA-629	Antibacterial	9-amino-acid peptide derivative of bactericidal/permeability-increasing protein	Impetigo	Phase III	Xoma
DRAMP 18155	DiaPep277	Unknown	DiaPep277 is a small, lyophilized powder containing 24 Amino-acids. It has proved in former studies that DiaPep277 can slow down beta cells destruction in the pancreas and therefore decelerate the progress of Diabetes.	Type 1 diabetes mellitus	Phase III	DeveloGen
DRAMP 18156	RDP58(Delmitide)	Unknown	Semisynthetic D-amino acid decapeptide derived from HLA class I B2702	Inflammatory bowel disease	Post Phase II	Genzyme; licensed to Procter&Gamble
DRAMP 18157	NVXT (Novexatin, NP213)	Antifugal(membrane)	cyclic cationic peptide derived from NovaBiotics arginine peptide platform	Onychomycosis (fungal nail infection)	Phase IIb	NovaBiotics
DRAMP 18158	PMX-30063(brilacidin)	Antibacterial	Defensin structural mimetic, non-peptide,small molecule/copolymer	Acute bacterial skin infections caused by Staphylococcusspp	Phase II	PolyMedix
DRAMP 18159	Oritavancin	Antibacterial	Oritavancin is a glycopeptide antibiotic used to treat acute bacterial skin and skin structure infections caused by susceptible Gram-positive bacteria. It was developed by The Medicines Company (acquired by Novartis).	Treatment of adult patients with acute bacterial skin and skin structure (including subcutaneous) infection.	In market	The Medicines Company

DRAMP 18161	OP-145 (AMP60.4 Ac)	Antibacterial	Synthetic 24-mer peptide derived from LL-37 for binding to lipopolysaccharides or lipoteichoic acid	Chronic suppurative otitis media (middle ear infections)	Phase II (Completed)	OctoPlus BV, Dr Reddy's Research and Developme nt BV
DRAMP 18162	BL2060	Unknown	Synthetic compound comprising fatty acid and lysine copolymers	Anti-infective	Lead optimization	BioLineRx (Jerusalem).
DRAMP 18163	Ghrelin	Unknown	Endogenous host-defence peptide,Synthetic construct	Airway inflammation, chronic respiratory infection and cystic fibrosis, cnacer	Phase II (Completed)	University of Miyazaki Third Department of Internal Medicine (Japan) and Papworth Hospital (UK).
DRAMP 18164	AP-214(Modi melanotid e)	Antibacterial	Synthetic derivative from HDP α-melanocyte-stimulating hormone	①Prevention of acute kidney injury after cardiac surgery; ② Treatment of sepsis.	Phase II (Completed)	Action Pharma A/S (Aarhus and Copenhagen , Denmark)
DRAMP 18165	CD-NP(Cende ritide)	Unknown	Synthetic chimeric 37-mer derived from combination of two natriuretic peptides	Acute decompensated failure (ADHF),Organ failure	Phase II	Nile Therapeutic s (United States, Minnesota).
DRAMP 18166	Vasoactiv e intestinal peptide (VIP; Endogeno us HDP)	Antibacterial	No more description	Acute Respiratory Distress Syndrome and Sepsis	Phase I	Stony Brook University and State University of New York (United States, New York).
DRAMP 18167	Ruminoco ccin C (Bacterioc in)	Antibacterial	Ruminococcus gnavus E1	Gastrointestinal tract infections; Stomach and intestine infections (Rat model; In-vivo)	Preclinical	Unknown
DRAMP 18168	Planospori cin (Bacterioc in)	Antibacterial	Planomonospora sp. DSM14920	Hospital-acquired infections; Multi-drug resistant strain (Murine model; In-vivo)	Preclinical	Unknown
DRAMP 18169	ESL5	Antibacterial	Enterococcus faecalis SL-5	Gastrointestinal tract infections; Stomach and intestine infections (Human model; In-vivo)	Preclinical	Unknown
DRAMP 18170	Colicin E1	Antibacterial	Escherichia coli H22	Gastrointestinal tract infections; Stomach and intestine infections (Murine model; In-vivo)	Preclinical	Unknown

	(Bacteriocin)					
DRAMP 18171	Lactocin 160 (Bacteriocin)	Antibacterial	Lactobacillus rhamnosus 160	Urogenital tract infections; Bacterial vaginosis	Preclinical	Unknown
DRAMP 18172	Bacteriocin OR-7	Antibacterial	Lactobacillus salivarius NRRL B-30514	Gastrointestinal tract infections; Campylobacter infection (Chicken model; In-vivo)	Preclinical	Unknown
DRAMP 18160	Omiganan (MBI-226/MX-226/CLS001)	Antibacterial	Pediocin PA-1 (Bacteriocin)	Pediococcus acidilactici UL5	Gastrointestinal tract infections; Stomach and intestine infections (Murine model; In-vivo)	Preclinical
DRAMP 18173	Pediocin PA-1 (Bacteriocin)	Antibacterial	Lactococcus lactis subsp	Urogenital tract infections; Spermicidal activity (Rabbit model; In-vivo)	Preclinical	Unknown
DRAMP 18174	Nisin A (Type A lantibiotic)	Antibacterial	Synthetic derivative of bactenecin from bovine neutrophils	Staphylococus aureusanti-infectives	Preclinical	Unknown
DRAMP 18175	Bac8c	Antibacterial(Membrane)	Frog (Rana ornativentris) skin	Staphylococus aureusanti-infectives	Preclinical	Unknown
DRAMP 18176	Temporin 10a	Antibacterial(membrane)	Frog (Leptodactylus syphax) skin	Staphylococus aureusanti-infectives	Preclinical	Unknown
DRAMP 18177	Syphaxin(SPX1-22)	Antibacterial	IDR-1 is a 13-amino acid peptide derived from bovine neutrophils that shows antimicrobial activity against MRSA, VRE, and other Gram-positive and Gram-negative pathogens.	Prevention of infections in the immune compromised	Phase I	Unknown
DRAMP 18178	IDR-1	Chemokine induction and reduction of pro-inflammatory cytokines (Scott et al., 2007)	Derivative of bactenecin from bovine neutrophils	Staphylococus aureusanti-infectives	Preclinical	Unknown
DRAMP 18179	IDR-1002	Chemokine induction and enhanced leukocyte recruitment	Asian Toad (Bufo bufo gargarizans) stomach	Staphylococus aureusanti-infectives	Preclinical	Unknown
DRAMP 18180	Buforin II	Antibacterial(nucleic acids)	Dusquetide is a synthetic, 5-amino acid peptide and Innate Defense Regulator (IDR), with immunomodulating, anti-inflammatory, anti-infective and anti-mucositis activities. Upon intravenous administration, dusquetide	Treatment for oral complications caused by radiation therapy for head and neck cancer.	Phase III	Soligenix

			binds to the ZZ domain of sequestosome-1, also called p62, and activates regulatory signaling transduction pathways involved in the modulation of the innate immune system, such as those mediated by mitogen-activated protein kinase (MAPK) p38 and CCAAT-enhancer-binding protein. This agent promotes monocyte and macrophage recruitment to, and accelerates healing in damaged and infected tissue; it suppresses inflammation through the regulation of the expression of multiple cytokines. This agent may prevent or decrease chemo- or radiotherapy-induced mucositis as well as other types of infection. p62, an intracellular adaptor protein that functions downstream of certain signaling receptors, plays a key role in the activation of the innate immune system.			
DRAMP 18181	DP178 (T20, Enfuvirtide & Fuzeon)	Anti-HIV	a synthetic AMP	Treatment of Adult and Adolescent Dental Subjects	Phase II	Lantibio
DRAMP 18182	Sifuvirtide (SFT)	Anti-HIV	Enfuvirtide is a 36 amino acid biomimetic peptide that is structurally similar to the HIV proteins that are responsible for the fusion of the virus to cell membranes and subsequent intracellular uptake. The first agent in the novel class of antiretroviral drugs called HIV fusion inhibitors, enfuvirtide works by inhibiting HIV-1 fusion with CD4 cells.	Human Immunodeficiency Virus (HIV) Infections; AIDS	Proved by FDA	Trimeris
DRAMP 18183	SB006 (M6)	Antibacterial	designed based on the 3D structure of the HIV-1 gp41 fusogenic core conformation	HIV fusion inhibitor;AIDS	Phase II	Unknown
DRAMP 20760	C16G2	Antibacterial	tetra-branched multimeric form of the linear sequence	Gram-negative infections	Preclinical	SpiderBiotech
DRAMP 20761	LTX-109	Antibacterial	LTX-109 is a broad-spectrum, fast-acting bactericidal antimicrobial drug for topical treatment, which causes membrane disruption and cell lysis.	Uncomplicated Gram positive skin infections, impetigo,and nasal colonization with S. aureus	Phase I/II	Lytix Biopharma AS
DRAMP 20773	Dusquetide(SGX942)	Antibacterial	synthetic by amino acid substitution of protegrin I.	Treatment of nosocomial pneumonia and ventilator-associated bacterial pneumonia (VABP)	Phase III(suspended, adverse events)	Polyphor Ltd.
DRAMP 20774	Murepavadin (POL7080)	Antibacterial	Omiganan (MBI-226), an analogue of indolicidin, has been proven to be capable of significantly reducing catheter colonization and microbiologically confirmed tunnel infections during catheterization	Rosacea, Acne vulgaris (II), Genital warts (II)	Phase III(catheter-related infections,Failure), Phase III(rosacea, completed), Phase II(atopic	Maruho Co., Ltd(developing), Cutanea Life Sciences, Inc.(developing),

					dermatitis (AD), usual type vulval intraepithelial neoplasia (uVIN), external genital warts, and acne vulgaris, completed), Phase III(facial seborrheic dermatitis, recruiting)	Mallinckrodt, Micrologix Biotech(Vancouver, BC, Canada)
DRAMP 28983	PL-5	Antibacterial	This product is a national category 1 anti-infective innovation category in China, and has obtained PCT US and Chinese invention patent authorizations. Suitable for skin and wound infections, especially for stubborn infectious diseases caused by drug-resistant bacteria, against traditional antibiotics, "super bacteria" Pseudomonas aeruginosa, and methicillin-resistant Staphylococcus aureus (MRSA) and multi-drug resistant Acinetobacter baumannii containing NDM-1 gene have very strong bactericidal advantages. Its preparation antimicrobial peptide PL-5 spray is the first antimicrobial peptide variety to enter clinical research in my country, and this product has also been supported by the National Health and Family Planning Commission's 12th and 13th Five-Year "Major New Drug Development" projects. Phase I clinical research has been completed, and Phase II clinical research is currently underway.	Skin wound infection	Phase IIIb	Changchun ProteLight Pharmaceutical & Biotechnology Co.
DRAMP 29315	Bacitracin	Antibacterial	Bacitracin A is a homodetic cyclic peptide consisting of (4R)-2-[(1S,2S)-1-amino-2-methylbutyl]-4,5-dihydro-1,3-thiazole-4-carboxylic acid attached head-to-tail to L-leucyl,D-glutamyl, L-lysyl, D-ornityl, L-isoleucyl, D-phenylalanyl, L-histidyl. D-aspartyl and L-asparaginyl residues coupled in sequence and cyclised by condensation of the side-chain amino group of the L-lysyl residue with the C-terminal carboxylic acid group. It is the major component of bacitracin. It has a role as an antibacterial agent and an antimicrobial agent. It is a homodetic cyclic peptide and a polypeptide.	prevent wound infections, treat pneumonia and empyema in infants, and to treat skin and eye infections.	In market	

DRAMP 29316	Surotomycin(MK-4261/CB-183,315)	Antibacterial	Surotomycin has been used in trials studying the treatment of Diarrhea and Clostridium Difficile Infection. It is a benzenebutanoic acid derivative patented by Cubist Pharmaceuticals, Inc. as antibacterial agents for the treatment of Gram-positive infections. Surotomycin has a fourfold greater in vitro potency than vancomycin against C. Difficile and other Gram-positive bacteria with minimal impact on the Gram-negative organisms of the intestinal microbiota. Surotomycin, given orally, has been shown to be highly effective against both initial and relapsing hamster Clostridium difficile-associated diarrhea, with a potency similar to vancomycin. Surotomycin is non-inferior to vancomycin and offers a promising alternative for the treatment and prevention of C. diff infection.	Treatment of Diarrhea and Clostridium Difficile Infection	Phase III	Cubist Pharmaceuticals Inc./Merck & Co. Inc.
DRAMP 29317	Colistin(polymyxin-E)	Antibacterial	Colistin is a polymyxin antibiotic used to treat bacterial infections caused by susceptible Gram negative bacteria.	Treatment of acute or chronic infections due to sensitive strains of certain gram-negative bacilli, particularly Pseudomonas aeruginosa.	In market	
DRAMP 29318	PXL01	Antibacterial	Synthetic macrocyclic 25-amino acid peptide derived from human lactoferricin	Prevention of post-surgical adhesion formation in hand surgery	Phase II/III	Promore Pharma (formerly Pergamum AB)
DRAMP 29319	HXP124	Antifungal	Plant defensin	Fungal nail infection (onychomycosis)	Phase II	Hexima
DRAMP 29320	DPK-060	unknown	Derived from kininogen, cationic random-coil peptide	Atopic dermatitis, Otitis externa	Phase II	DermaGen AB/Pergamum AB/Karolinska Development AB
DRAMP 29321	Lotilibcin(WAP-8294A2)	Antibacterial	Lipodepsipeptide	Methicillin-resistant S. aureus	Phase I/II	aRigen Pharmaceuticals, Green Cross Corporation
DRAMP 29322	NVB302	Antifungal	Synthetic type B lantibiotic	C. difficile infections	Phase I	Novacta Biosystems Ltd.
DRAMP 29323	EA360	unknown	Linear tetrapeptide, derived from human chorionic gonadotropin	Systemic inflammatory response and renal function	Phase IIa/b current	Exponential Biotherapies
DRAMP 29324	Friulimicin B	Antibacterial	Friulimicin B is a naturally occurring antibiotic produced by a micro-organism, *Actinoplanes friuliensis*. It shows strong cidal activity against a number of Gram-	Pneumonia, staphylococcal skin infections	Phase I	MerLion/Pharmaceuticals

			positive pathogens which commonly cause serious infections in hospital patients and which are becoming more routinely acquired in the community.			
DRAMP 29325	TD-1792(Cefilavancin)	Antibacterial	Cefilavancin is a covalently-linked glycopeptide-cephalosporin (beta-lactam) heterodimer antibiotic that exhibits substantially greater activity than its component parts against Gram-positive bacteria.	Gram-positive infections, Skin and soft tissue infections	Phase III	GlaxoSmith Kline Co., Theravance Biopharma Inc., R-Pharm
DRAMP 29326	LTX-315(Oncopore, Ruxotemitide)	Anticancer	Ruxotemitide is a peptide derived from human lactoferrin, with potential lytic and immunostimulating activities.	Solid tumors	Phase I	Lytix Biopharma
DRAMP 29327	ANG1005 (Paclitaxel trevatide)	Anticancer	Paclitaxel Trevatide is a peptide-drug conjugate containing the taxane paclitaxel covalently linked to the proprietary 19 amino acid peptide angiopep-2, in a 3:1 ratio, with potential antineoplastic activity.	Breast cancer, brain metastases	Phase II	Angiochem Inc.
DRAMP 29328	Polymyxin B	Antibacterial	Polymyxin B is a polymyxin antibiotic used to treat a wide variety of infections in the body.They are basic polypeptides of about eight amino acids and have cationic detergent action on cell membranes.	Treatment of infections of the urinary tract, meninges, and blood stream, caused by susceptible strains of Pseudomonas aeruginosa	In market	Unknown
DRAMP 29329	Tyrothricin	Antibacterial, Antifungal	A polypeptide antibiotic mixture obtained from Bacillus brevis. It consists of a mixture of three tyrocidines (60%) and several gramicidins (20%) and is very toxic to blood, liver, kidneys, meninges, and the olfactory apparatus. It is used topically.Tyrothricin is a topical antibiotic with broad spectrum activity against Gram positive bacteria and some fungal infections.	Treatment of infected skin and infected oropharyngeal mucous membranes	In market	Unknown
DRAMP 29330	Vancomycin	Antubacterial	Vancomycin is a glycopeptide antibiotic used to treat severe but susceptible bacterial infections such as MRSA (methicillin-resistant Staphylococcus aureus) infections.	Treatment of septicemia, infective endocarditis, skin and skin structure infections, bone infections, and lower respiratory tract infections.	In market	Unknown
DRAMP 29331	Onc72	Antibacterial	derived from Oncocin	Treatment of antibiotic-susceptible K. pneumoniae	Preclinical	Unknown
DRAMP 29332	Gramicidin S	Antibacterial	Cyclic peptide biosynthesized from gramicidin in Bacillus brevis; comprises two identical pentapeptides coupled head to tail.	Potent against gram-negative and gram-positive bacteria and fungi restricted use as spermicide and to treat genital ulcers caused by STD	In market	Unknown
DRAMP 29333	Gramicidin D	Antibacterial	Gramcidin D is a heterogeneous mixture of three antibiotic compounds, gramicidins A, B and C, making up 80%, 6%, and 14% respectively all of which are obtained from the soil bacterial species Bacillus brevis and called collectively gramicidin D. Gramcidins are 15 residue peptides with alternating D	Skin lesions, surface wounds and eye infections	In market	Unknown

	and L amino acids, which assemble inside of the hydrophobic interior of the cellular lipid bilayer to form a β-helix. Active against most Gram-positive bacteria and some Gram-negative organisms, Gramicidin D is used primarily as a topical antibiotic and is also found in Polysporin ophthalmic solution.	
